# Ileocecal Duplication Cyst Masked by Appendicitis: A Surgeon's Eternal Dilemma

**DOI:** 10.7759/cureus.62829

**Published:** 2024-06-21

**Authors:** Anuradha S Dnyanmote, Kuldip. Patil, Rushi Kanani, Vidita Modi

**Affiliations:** 1 Surgery, Dr DY Patil Medical College, Hospital and Research Centre, Pune, IND; 2 General Surgery, Dr DY Patil Medical College, Hospital and Research Centre, Pune, IND; 3 General Surgery, Dr DY Patil Medical College, Hospital and Research Centre, PUNE, IND

**Keywords:** intestinal duplication, ileocecal, appendicular perforation, appendicular mass, appendicits

## Abstract

Alimentary tract duplications are uncommon abnormalities. They are mostly found in the terminal ileum, and most develop symptoms before the age of two. Abdominal mass, intestinal blockage, intussusception, rectal hemorrhage, and abdominal pain are possible presenting signs. Intra-abdominal duplications are typically discovered during surgical examinations of the problems; preoperative diagnosis is typically challenging. Our unusual adult male patient, age 32, had an asymptomatic ileal-caecal junction duplication cyst that was linked to a non-complicated acute appendicitis.

## Introduction

It is possible for duplications, which are extremely uncommon gastrointestinal tract malformations, to occur in any portion of the gastrointestinal system, ranging from the oral cavity down to the anus. The ileocecal region, the terminal ileum, and the esophagus are the areas in which they are found most commonly. Seventy-three percent of duplicates will exhibit symptoms before the age of one, and 85% will do so before the age of two. The rest might not manifest until they are much older or even until they are adults [1,2]. In most cases, symptoms include an intra-abdominal mass, bleeding from the rectal area, obstruction of the intestinal tract, abdominal pain, and intussusception [3]. It is typically challenging to make a preoperative diagnosis of GI duplications. Imaging procedures using radiation might not be sufficient to make a conclusive diagnosis.

## Case presentation

A 32-year-old adult male who did not have any co-morbidities was sent to our surgical department as an outpatient, complaining of abdominal pain, nausea, and vomiting for the past 10 days. The pain was predominantly in the right lower abdomen, sudden in onset, gradually progressive, with no obvious relieving factors. The pain didn’t aggravate with micturition or with each passage of bowel movements. The patient also had a history of fever for three days, not associated with chills and rigors. There was no history of trauma, drug abuse, or any surgical intervention in the abdominal region in the past.

On examination, the patient was afebrile on examination with stable hemodynamics. The patient exhibited tenderness and guarding in the right lower quadrant when being examined physically.

According to the results of the laboratory tests, the patient had leukocytosis (WBC: 13.51 109/L) and an elevated level of C-reactive protein (33 mg/dl). The X-ray of the abdomen did not reveal any abnormalities that may be considered pathologic. An ultrasound abdomen and pelvis were suggestive of a blind-ending, tubular, anechoic, aperistaltic structure measuring 9.7 mm in RIF near the ileocecal junction. Additionally, there was a cystic lesion measuring 26 x 30 mm near the distal ileum. A working diagnosis of appendicular mucocoele was made, and the patient was worked up for surgery.

CECT (contrast-enhanced computed tomography) abdomen and pelvis confirmed the USG (ultrasound sonography) findings. As suggested by the ultrasound, there was a well-defined fluid density measuring 30 x 25 mm noted arising from the distal ileum without signs of inflammation or perforation.

**Figure 1:**
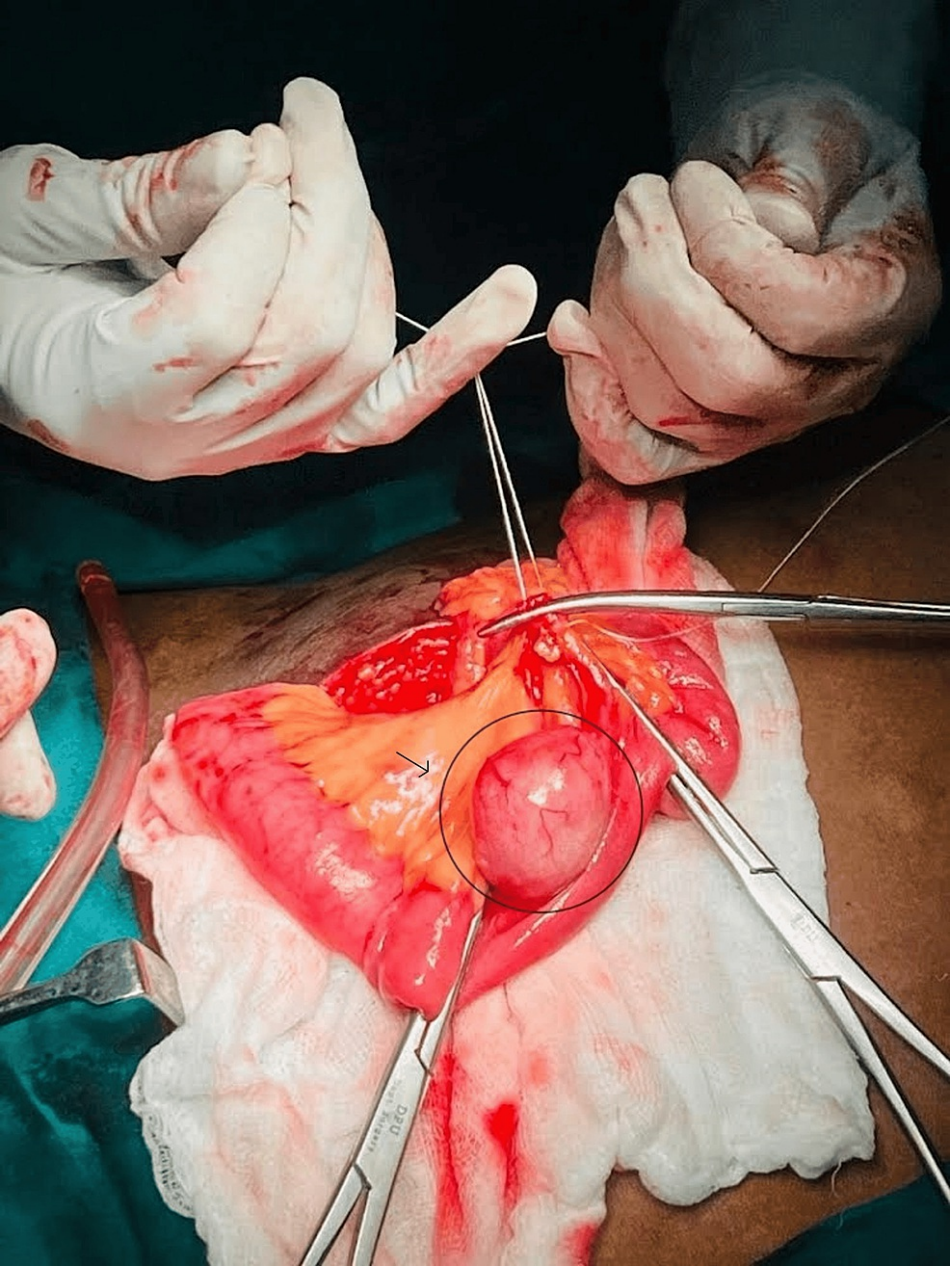
3 x 4 cm mesenteric cyst was identified approximately 20 cm from ileocecal junction

The patient underwent a diagnostic laparoscopy, which revealed a 7 cm caecal appendix, inflamed and adherent to the peritoneum. An appendectomy was performed for the patient. On further inspection of the bowel, a 3 x 4 cm mesenteric cyst was identified approximately 20 cm from the ileocecal junction (Figure 1).

**Figure 2:**
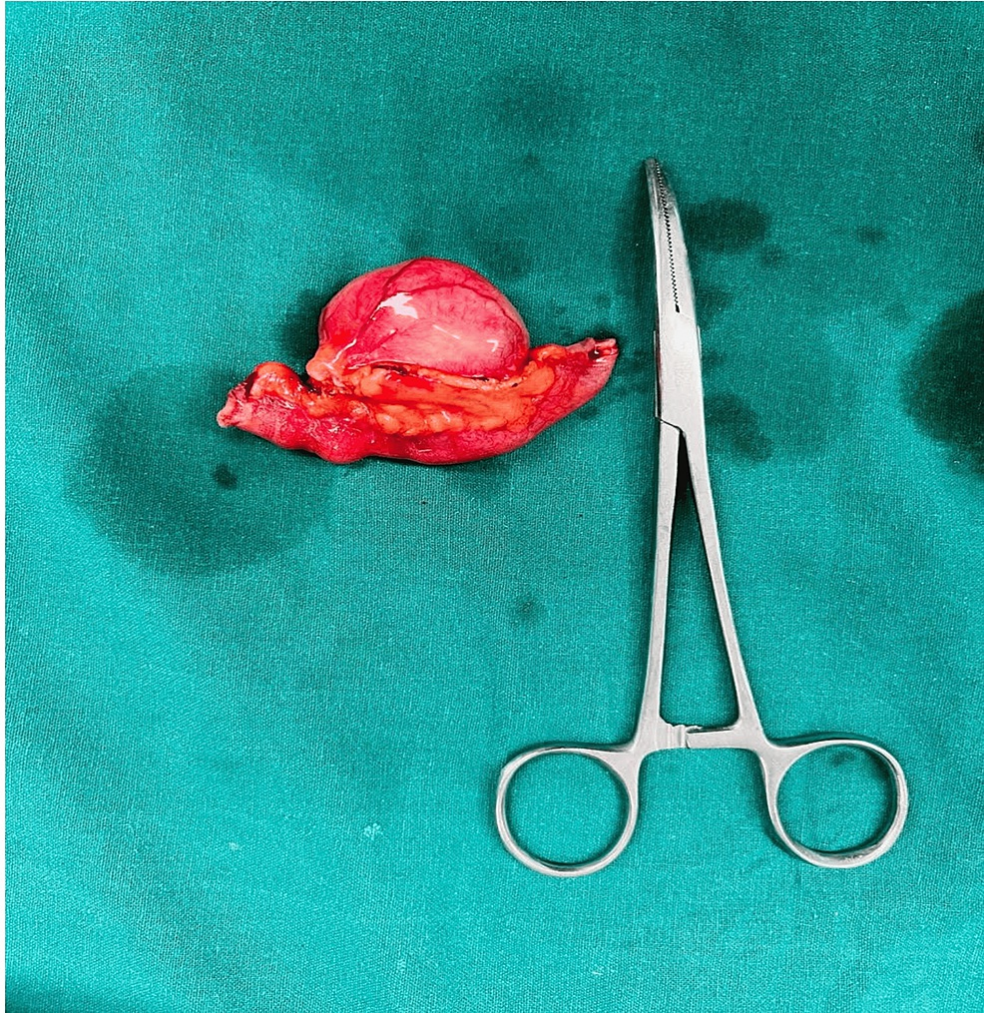
1 x 1 cm mesenteric lymph node was excised and sent for histopathology

The position of the cystic lesion with the adjacent 2 cm bowel was clamped and excised. An end-to-end anastomosis was fashioned in two layers. 1 x 1 cm mesenteric lymph node was excised and sent for histopathology (Figure 2). The abdomen was then closed in layers, and the patient was shifted to a postoperative ward with stable hemodynamics.

On the third postoperative day, the patient was started on enteral nutrition, and we were able to discharge the patient without any difficulties. The specimens were examined using histopathology, which revealed appendicitis with local peritonitis as well as a duplication cyst walled with ileal mucosa and free of any ectopic tissue.

## Discussion

Rare congenital defects, gastrointestinal duplications can be detected from mouth to anus. Approximately 1:4500-1:10000 people have it [1,2]. Persistence of fetal intestine diverticula, defect in primitive gut solid phase recanalization, partial twinning, and split notochord theory are possible causes. Although its cause is unknown, “the intrauterine vascular accident theory” is the most widely recognized [3]. This developmental defect is most frequent in the ileum but can also occur in the esophagus, duodenum, and rectum. Cystic, tubular, and intramural duplication are macroscopic. Most cases are cystic duplication [4,5]. Duplication cysts feature a smooth muscle layer and are bordered with neighboring alimentary tract mucosa, which may contain ectopic gastric or pancreatic tissue [6,7]. Our cystic duplication was found in the ileum, where it is most common, and was lined by the ileal intestinal epithelium, a few tubular structures beneath the mucosa, and a common muscle layer with the adjacent intestinal tissue. No heterotopic tissue was found in our case.

As in our patient, some duplications remain asymptomatic before age two. Duplication cyst symptoms depend on location, size, and heterotopic mucosa. Internal abdominal mass, rectal hemorrhage, intestinal blockage, and abdominal pain are typical [4,8]. Volvulus, intussusception-related intestinal obstruction, or ectopic gastric mucosa-related ulceration can induce abdominal pain. In our case, abdominal discomfort, nausea, and vomiting were associated with appendicitis because the ileal duplication cyst was simple as it was asymptomatic and accidental in finding. Adult ileal duplication cyst adenocarcinoma is rare [3,9].

Due to their diverse symptoms, duplication cysts are hard to diagnose preoperatively. US and barium contrast examinations are the most used diagnostic imaging modalities. Fewer people need CT and MRI. Today, prenatal imaging methods diagnose many duplication cysts [8]. US duplication cysts have echogenic inner mucosal and hypoechoic outside muscle layers [10]. Preoperative US imaging showed an inflamed appendix over 9 mm in diameter, but it was a duplication cyst.

## Conclusions

Congenital intestinal duplication cysts are rare. Most occurrences are symptomatic and detected before age two, although they can also occur asymptomatically and incidentally with acute appendicitis, as in this case. Even when found inadvertently, cystic duplications should be surgically removed to avoid volvulus, blockage, invagination, and hemorrhage. The complications associated with it could be life-threatening.
